# The metaphyseal bone defect in distal radius fractures and its implication on trabecular remodeling—a histomorphometric study (case series)

**DOI:** 10.1186/s13018-015-0205-9

**Published:** 2015-05-09

**Authors:** Martin Lutz, Roland Steck, Ingrid Sitte, Michael Rieger, Michael Schuetz, Thomas Klestil

**Affiliations:** Department for Trauma Surgery, LK Baden Mödling, Sr. M. Restituta Gasse 12, 2340 Mödling, Austria; Institute of Health and Biomedical Innovation, Queensland University of Technology Brisbane, 60 Musk Avenue, Kelvin Grove QLD, 4059 Brisbane, Australia; Department for Trauma Surgery, Medical University Innsbruck, Anichstrasse 35, 6020 Innsbruck, Austria; Department for Radiology General Hospital Hall in Tirol, Milser Strasse 10, 6060 Hall in Tirol, Austria

**Keywords:** Distal radius fracture, Bone void, Trabecular ultrastructure, Histomorphometry

## Abstract

**Background:**

The invention of the locking plate technology leads to alterations of treatment strategies at metaphyseal fracture sites with the concept of spontaneous remodeling of trabecular bone voids. Whereas trabecular regeneration has been proven in experimental animal studies, no histologic data exist on human fracture healing with special emphasis on bone voids.

**Methods:**

In order to qualify the trabecular bone remodeling capacity in vivo, bone specimens from the metaphyseal bone void were analyzed 14 months after trauma using quantitative histomorphometry. Twenty-five patients with an unstable dorsally displaced distal radius fracture were fixed with a palmar locking plate without additional bone graft or substitute. At implant removal, specimens from the previous compression void were harvested with a trephine in a volar-dorsal direction. In 16 patients, histomorphometric analysis could be performed, comparing the dorsal trabecular network with the volar, non-compressed ultrastructure.

**Results:**

Significant differences for bone volume/total volume (BV/TV), trabecular number (TbN) and trabecular separation (TbSp), but not for trabecular thickness (TbTh) and osteoid volume/total volume (OV/TV), were detected. Neither patient age, defect size nor gender had a significant influence on bone remodeling.

**Conclusions:**

The results of this study indicate that trabecular bone remodeling does not lead to pre-trauma bone quality in metaphyseal bone compression voids following reduction and application of a locking plate.

## Background

During the last decade, substantial progress has been made towards the understanding of cortical bone fracture healing. Improved implant design and treatment strategies like percutaneous plate fixation with semi-rigid constructs demonstrated to preserve the blood supply of bony fragments and enhance callus formation, compared with more traditional techniques of absolute stability.

However, with the demographic shift towards an ageing population and the corresponding increase of fractures in metaphyseal regions, research focus has been directed towards trabecular bone fracture healing. In particular, recent research projects investigate the impact of interfragmentary instability on trabecular bone fracture healing, as well as the bone regeneration capacity in metaphyseal defects [1,2].

A particular challenge is the treatment of metaphyseal bone defects, which occur after the stabilization and anatomical reconstruction of compression fractures, e.g. at the distal radius, with internal or external implants. For many years, metaphyseal bone defects were commonly filled with cancellous or corticocancellous bone autografts harvested from the iliac crest. Bone grafts have been proven to enhance the stability of the construct and to accelerate bone healing [3,4]. These techniques have been applied with good outcomes reported for both upper and lower extremity fractures, such as the distal radius, the phalangeal fractures, the proximal and distal tibia, as well as the calcaneus [5-9].

Due to the increased stability achieved with locking screw and plate technology, the use of bone grafts has been in decline [10]. These new devices enable reliable bridging of metaphyseal bone defects of variable size and enable rigid fixation of individual articular fragments in an anatomical position. Bony healing without loss of reduction has since been reported for such fractures, and even the restoration of defects following opening osteotomies without the application of additional bone grafts or bone graft substitutes has been described [11].

This clinical experience suggests a certain trabecular bone defect healing capacity, which has also been confirmed in several animal studies. However, these studies have also shown that the regeneration potential for trabecular bone defects is limited [12-14]. While small defects heal spontaneously, it has been demonstrated that from a critical defect size upwards, stable fixation with implants alone is not sufficient to maintain reduction [2].

The aim of the present study was therefore to determine the trabecular bone regeneration potential in clinical cases of distal radius fractures. Histomorphometric analysis of specimens from the initial metaphyseal bone defect was performed and compared with the volar less traumatized trabecular ultrastructure, at implant removal.

### Patients and method

Twenty-five patients with a dorsally displaced distal radius fracture were included in this study. All fractures have been fixed surgically in our institution between 2004 and 2008. Mean patient age at the time of injury was 54 years (min 18/max 75). Six men and 19 women were enrolled, and the fracture distribution according the AO classification scheme was as follows:

- A2 4
- A3 3
- C1 1
- C2 10
- C3 7

Fracture management included the initial closed reduction under local anaesthesia, injected into the fracture gap and application of a cast. Computer tomography (CT) was used, to determine articular involvement and to calculate the volume of the metaphyseal compression defect according to the method described by Flinkkila and coworkers [15].

**Figure 1:**
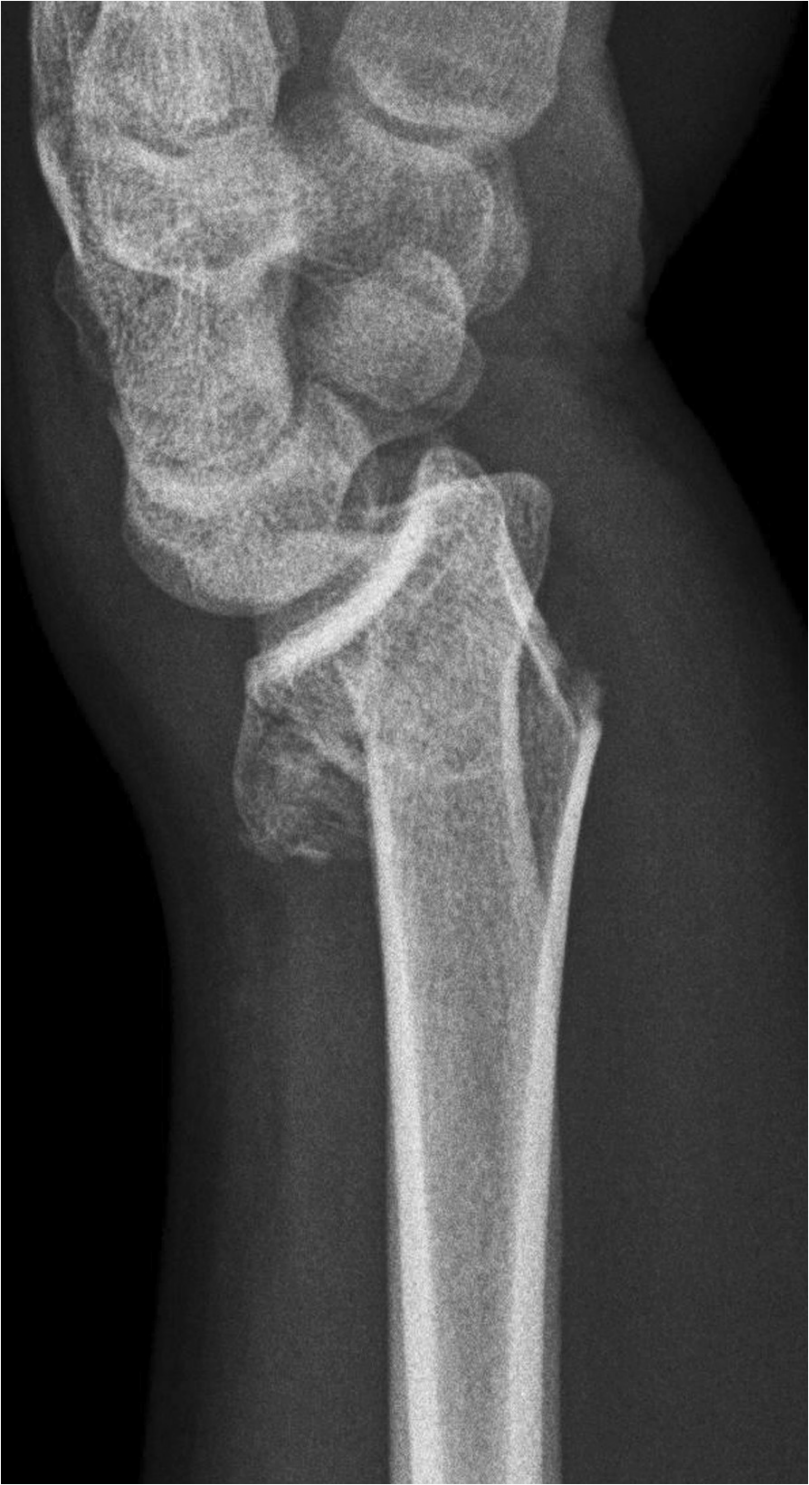
Unstable distal radius fracture; X-ray lateral view.

**Figure 2:**
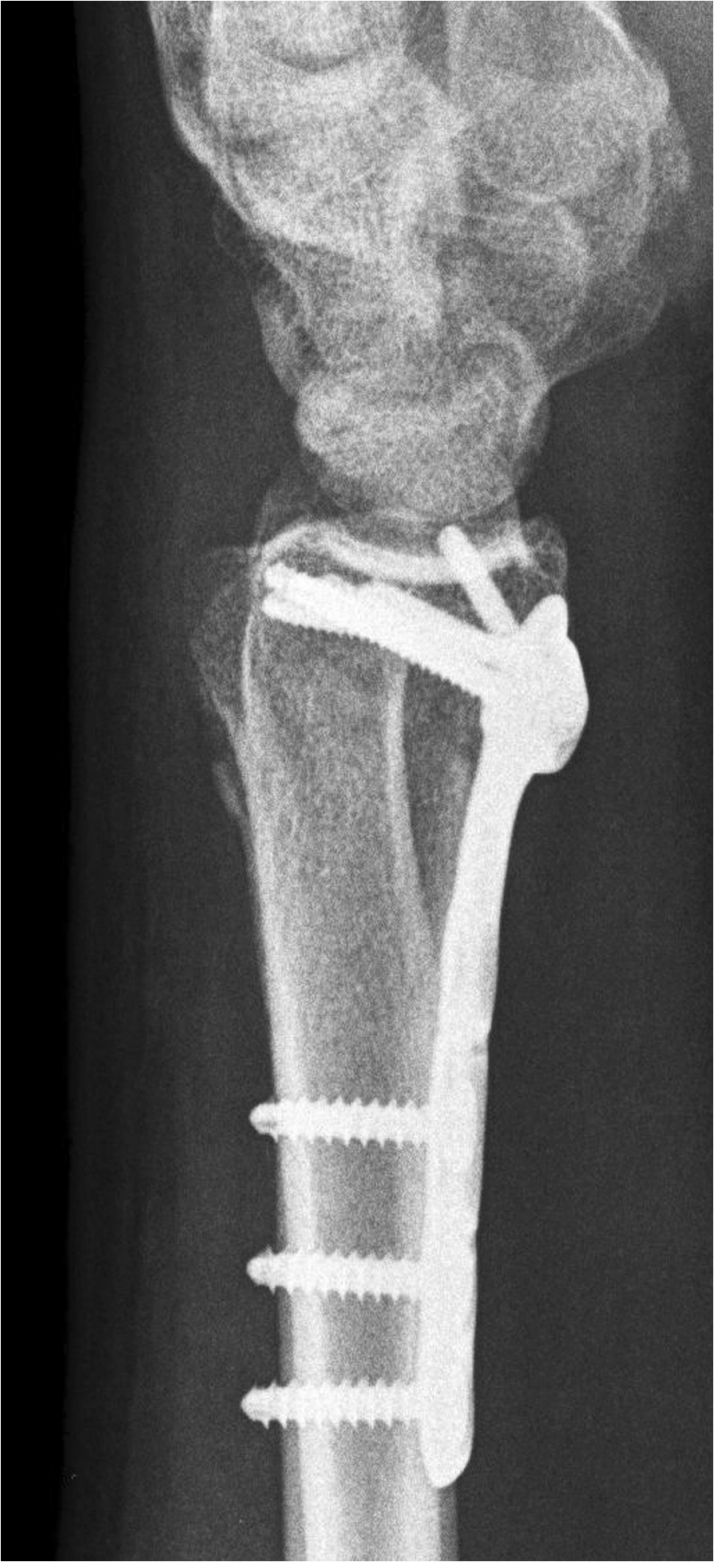
Lateral view demonstrating healing of the fracture with appropriate alignment after fracture fixation.

Thereafter, a locking plate was applied using a volar approach. Reduction of the articular and metaphyseal fracture fragments was confirmed with an image intensifier. In none of these cases, bone graft or bone graft substitutes were used for filling the defect. The postoperative regimen included a forearm splint for 3 weeks, followed by a physiotherapy programme (Figures 1 and 2).

One inclusion criterion for this particular study was radiologic healing of the fracture without loss of reduction. Neither ulnar variance nor dorsal tilt and radial inclination changed from postoperative till implant removal.

**Figure 3:**
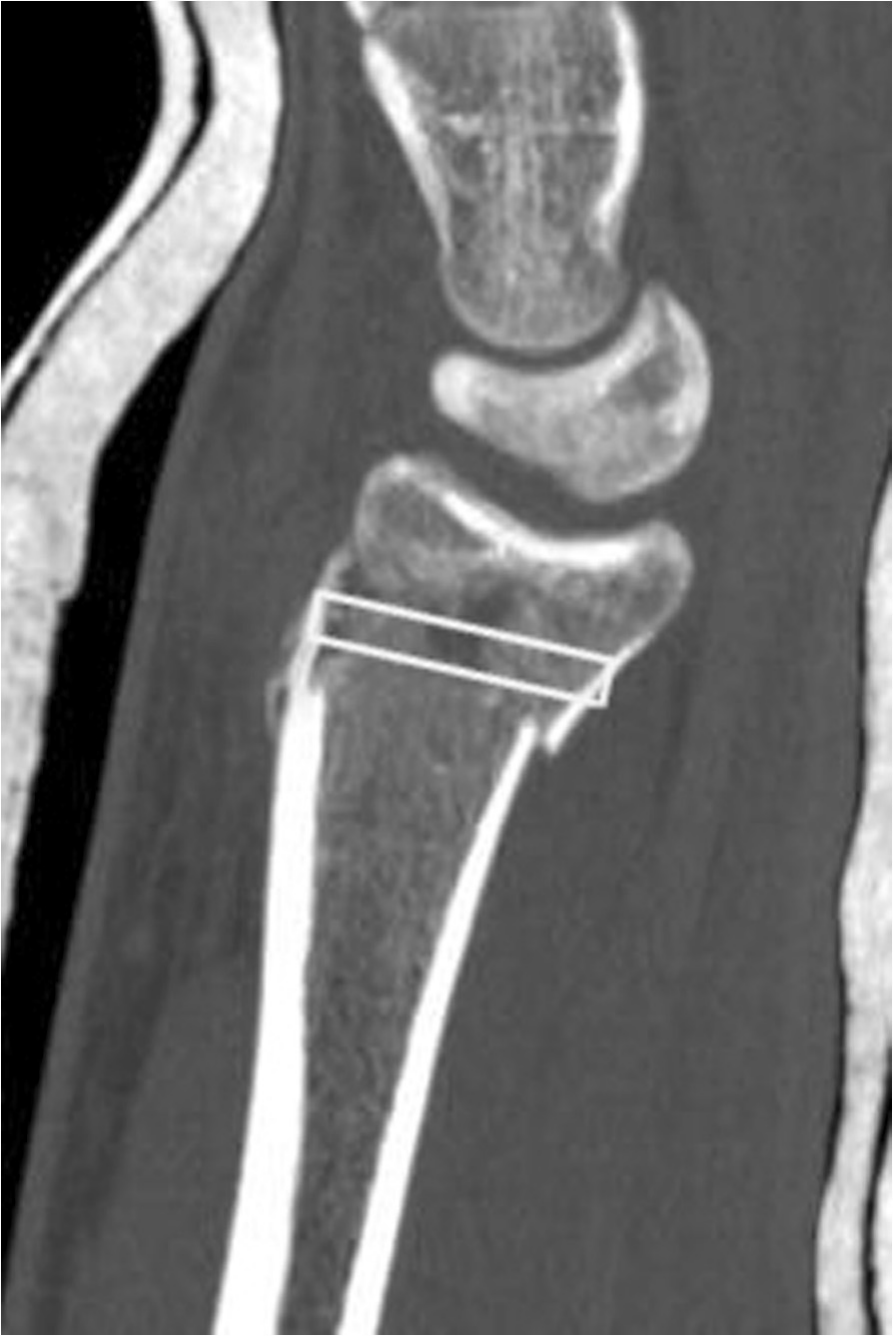
Lateral and axial CT scan after closed reduction and initial plaster fixation of the fracture: the diagram shows the orientation of the trephine insertion through the initial compression void at implant removal.

**Figure 4:**
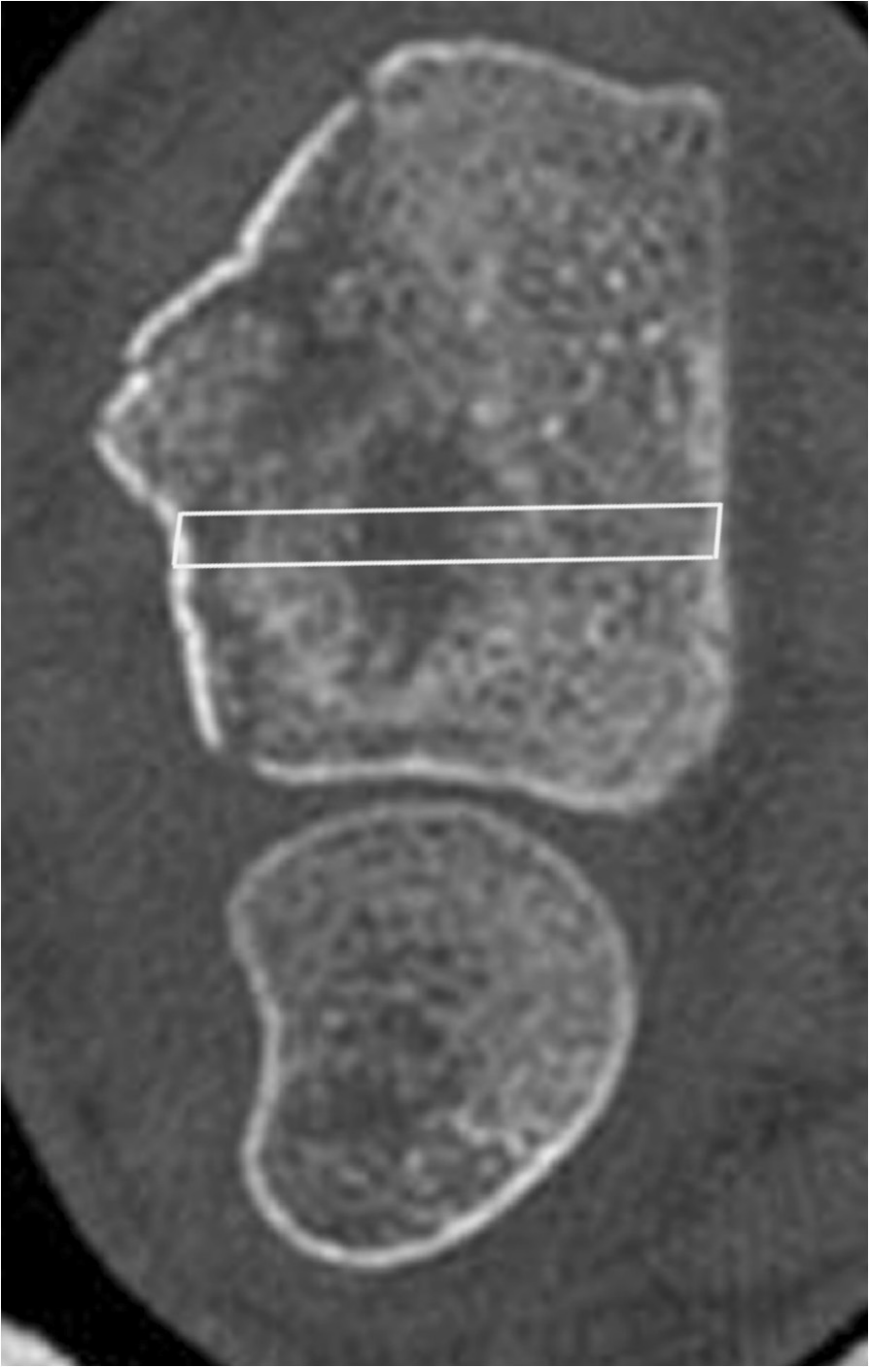
Lateral and axial CT scan after closed reduction and initial plaster fixation of the fracture: the diagram shows the orientation of the trephine insertion through the initial compression void at implant removal.

After bony healing, confirmed by conventional X-rays in two planes, the fixation implants were removed 14 months (min 6/max 30) after injury. During this surgery, bone biopsies were harvested using a trephine with a core diameter of 2 mm (Medical Device Technologies Inc; FL, USA). The volar cortex was opened with an awl, in order to avoid compression fractures of the trabecular network during extraction of the biopsy. The trephine was inserted from volar to dorsal aiming to sample the region of the metaphyseal compression defect. An image intensifier was used intraoperatively to guide the trephine into the previous compression zone. The distance between the distal articular surface of the radius and the compression void on the sagittal sections of the CT scan as well as the carpal bones on the anterior-posterior sections were chosen for orientation. This information enables the localization of the bone void on the postoperative X-ray with respect to the screws in the T-bar of the plate. With this in mind, the entrance point of the trephine was chosen and the intraosseous direction was checked with the image intensifier (Figures 3 and 4).

After harvesting, the specimens were fixed in 4% buffered paraformaldehyde, dehydrated in increasing ethanol concentrations and then embedded in methylmethacrylate for further processing for undecalcified histology.

Serial sections of 5 μm in thickness in a volar to dorsal orientation were produced using a microtome (Polycut, Reichert Jung) and the most central parts of the biopsy chosen for histomorphometric analysis. The sections were stained by Goldner trichrome indicating calcified trabecular bone green and osteoid red. They were then digitized with a Leica microscope DM 6000B (Leica Headquarters, Wetzlar, D-35578, Germany) at a magnification of ×20. Measurements were performed on binarized images of histological sections using National Institutes of Health Image J 1.42q (NIH, Bethesda, MD, USA) software.

**Figure 5:**
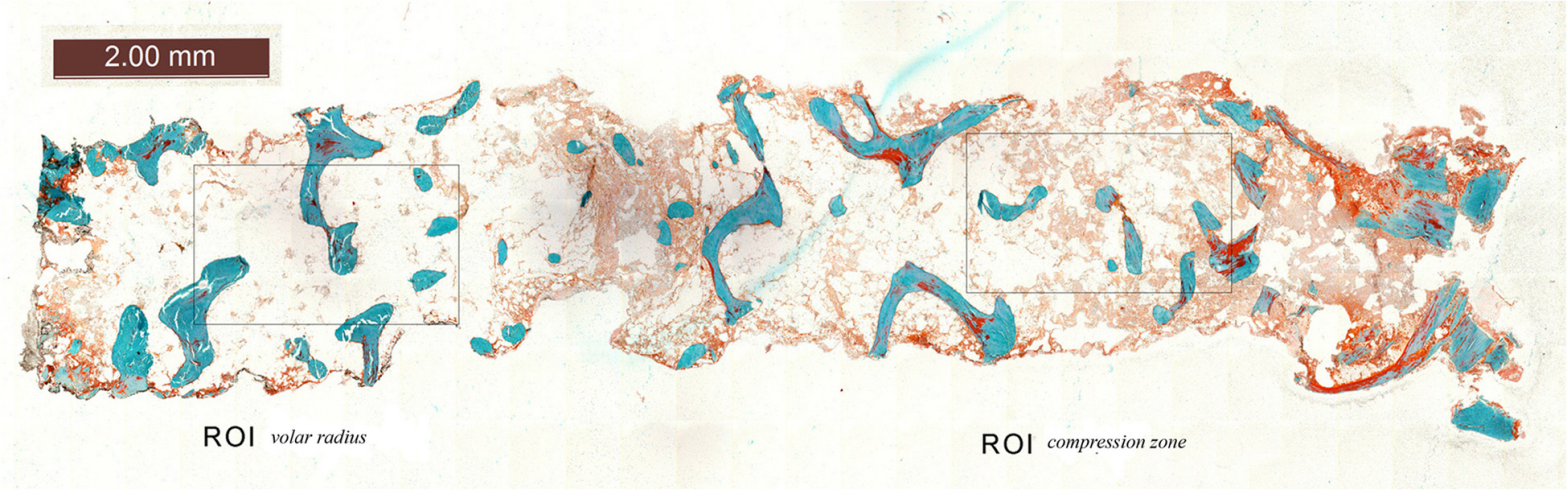
Histological section at implant removal with ROI V and ROI C depicting the volar and compression zone area, respectively.

To compare the trabecular structure of the dorsal comminution zone with the volar, less affected bony network, two regions of interests (ROI) of the same size (2.5 × 1.5 mm) were chosen. The first ROI was located in the dorsal area of the section, representing the compression defect zone, which was determined from the post-reduction CT scan. The second ROI was located in the volar third of the specimens, where the trabecular network is usually preserved (Figure 5).

In both ROIs, the trabecular bone contours were manually traced and the histomorphometric parameters BV/TV (bone volume/total volume), TbTh (trabecular thickness), TbSp (trabecular separation) and TbN (trabecular number) were calculated according to Parfitt et al. [16]. In addition, the osteoid area (stained bright red) was calculated after the images were passed through a filter and a threshold was set (OV/TV, osteoid volume/total volume).

Statistical analysis was performed using SPSS version 17.0 (SPSS, Inc., Chicago, IL, USA). Paired *t*-tests were performed to compare histomorphometric parameters from the two different regions. Differences with *p* < 0.05 were considered significant. Pearson correlations were performed to test for dependencies between age, defect size or gender and any of the histomorphometric parameters. All values are represented as mean ± standard deviation.

### Ethics

This study was conducted in accordance with the declaration of Helsinki (1996 Revision) and was approved by the institutional ethics committee of the Medical University of Innsbruck, Austria. Specific informed consent for participation in the study was obtained from all patients individually.

## Results

The average volume of the compression zone defect, as determined from post-reduction CT images, was 0.61 ± 0.42 mm^3^. Nine specimens were not suitable for quantitative analysis due to destruction of the trabecular network during the harvesting procedure, or due to an overall sample length of less than 8 mm, therefore representing only a fraction of the radial diameter at the fracture level. For the remaining 16 specimens, the values for the microarchitectural parameters BV/TV, TbN, TbTh, TbSp and OV/TV showed a relatively wide distribution throughout the study group (Table 1). Statistically significant differences between the two regions of interest were detected for the microarchitectural parameters BV/TV, TbN and TbSp, indicating that the bone quality in the dorsal defect zone had not recovered to pre-trauma values of the volar aspect. No statistical significant difference was found for the parameters TbTh and OV/TV (Table 2).

**Table 1:** **Mean and standard deviation of ultrastructural parameters in volar and dorsal ROIs of the distal radius at implant removal; sample size of the specimen and initial metaphyseal defect size**

	**ROI volar radius (SD)**	**ROI compression zone (SD)**	**Confidence interval of difference (** ***p*** **)**	**mm (SD)**	**mm** ^**3**^ **(SD)**
BV/TV	16.83 (3.23)	13.12 (5.76)	0.013		
TbN	1.63 (0.28)	1.34 (0.45)	0.036		
TbTh	104.69 (18.59)	95.35 (18.66)	0.099		
TbSp	526.63 (97.73)	763.43 (404.57)	0.036		
OV/TV	2.09 (1.10)	1.92 (1.08)	0.460		
Sample length				13.14 (2.69)	
Defect size					0.61 (0.42)

**Table 2:** **Ratio of individual ultrastructural parameters between compression zone (C) and volar ROIs (V) at implant removal; sample size of the specimen and metaphyseal defect size**

**Initials**	**Ratio C vs. V BV/TV**	**Ratio C vs. V TbN**	**Ratio C vs. V TbTh**	**Ratio V vs. C TbSp**	**Ratio C vs. V OV/TV**	**Age**	**Specimens length (mm)**	**Defect size (mm** ^**3**^ **)**
B G l	1.22	1.34	0.91	1.4	1.57	47	15.75	0.337
B G r	0.97	0.95	1.02	0.94	1.59	47	18.84	1.267
B E	0.37	0.64	0.58	0.56	0.58	71	12.23	0.178
B R	1.13	0.94	1.19	0.97	0.44	63	8.63	0.316
E M	0.41	0.5	0.84	0.43	0.98	65	9.02	0.299
H G	0.69	0.67	1.03	0.65	0.69	52	11.55	0.167
K S	0.37	0.27	1.38	0.24	0.92	64	12.47	1.415
K M	1.19	1.07	1.1	1.1	0.69	67	15.84	1.208
K M	0.45	0.63	0.71	0.57	1.36	68	11.81	0.187
M H	1.11	1.26	0.88	1.31	1.11	58	12.03	0.511
L J	0.92	0.91	1.01	0.89	0.86	23	11.95	0.264
N H	0.89	1.02	0.87	0.99	1.29	62	15.97	0.696
P S	0.73	0.98	0.74	0.94	1.17	21	14.04	0.38
O W	0.33	0.48	0.69	0.43	1.48	61	13.93	0.65
P E	0.94	0.86	1.09	0.84	0.48	62	15.03	0.971
R C	0.75	0.91	0.82	0.87	0.76	75	11.21	0.92

No significant correlations were found between patient age, gender, defect size and any of the microarchitectural parameters.

## Discussion

In this study, we compared the trabecular bone structure between the dorsal metaphyseal compression zone and the volar, less affected region after reconstruction and internal fixation of a distal radius compression fracture. We found that the bone quality, as defined by histomorphometric parameters for trabecular bone, was significantly different in the two regions after fracture healing and at the time of implant removal. These results clearly demonstrate the limited regenerative potential of trabecular bone in metaphyseal distal radius compression fractures.

To our knowledge, this represents the first demonstration of this phenomenon in the clinically relevant anatomical location of the distal radius with a high incidence of metaphyseal compression fractures. However, these findings confirm the results of previous studies at other anatomical sites. For instance, Gerich et al. [17] demonstrated in highly comminuted periarticular tibia head fractures a subsidence of articular fragments as well as metaphyseal misalignment after healing. The results of our study also correspond with the outcomes of recent animal studies on metaphyseal bone healing. These experimental studies have demonstrated that there is a so-called critical size for trabecular bone defects: While small defects healed spontaneously, the body’s regeneration processes were unable to repair defects above a certain critical size. Insufficient bone quality and fibrous tissue were detected in larger drill hole defects in a murine model [12].

Despite the reduced bone quality in the previous compression zone, trabecular bone healing around this area can be expected in most cases at the distal radius, as solid radiographic healing was confirmed by the study of Figl and coworkers [11]. After open reduction and application of a locking plate, no loss of reduction was observed in patients older than 75 years at a follow-up of 13 months.

The capability to achieve bony healing in periarticular fractures with metaphyseal bone defects is of major importance, because the posttraumatic axial alignment and joint congruency rely on this healing capacity. The concept of spontaneous trabecular remodeling became popular with the introduction of locking plates. However, clinically derived ultrastructural data on metaphyseal bone regeneration in the literature that would confirm this spontaneous trabecular bone remodeling are scarce [18].

In our opinion, the dorsally dislocated distal radius fracture is an ideal anatomical location for the study of trabecular bone regeneration, due to the high frequency of fractures in this location and the easy accessibility for detailed examination. The fracture is characterized by a dorsal comminution zone of variable size and a split fracture on the volar side with minor trabecular deterioration. After indirect anatomical reduction and fracture stabilization the dorsal compression zone is opened up and forms a trabecular bone void which can be defined according the technique of Flinkkila and coworkers [15].

Harvesting of bone biopsies from this location therefore allows for the comparison of two regions of interest from the dorsal and volar aspects of the radius, in order to analyze the ultrastructural differences in trabecular bone architecture.

During recent years, a detailed understanding of the trabecular ultrastructure of the distal radius has been gained with further insights into age- and gender-related changes. Quantitative CT studies have shown that bone quality and quantity is highest in the distal subchondral area and decreases towards the diaphysis [19]. Age-dependent changes result in a global deterioration of the ultrastructure from solid, plate-like trabeculae towards fragile, rod-like trabeculae. However, the general gradient of decreasing bone quality from distal to proximal remains.

In a micro CT study of anatomic distal radius specimens, which is nowadays accepted as the gold standard for three-dimensional trabecular ultrastructure analysis, Braunstein and coworkers detected no significant difference between the anterior and posterior trabecular networks in the same coronal plane. This is the basis for the present study and the rational for comparison of the anterior area with the previous compression void [20]. This provided the baseline for our comparison of the volar trabecular structure with the dorsal comminution zone following distal radius fractures. Consequently, the trephine was inserted perpendicular to the radius shaft using an image intensifier, aiming into the previous compression zone. This technique provided samples of one coronal plane, allowing comparable quantitative analysis.

In contrast, Sode and coworkers used pQCT to analyze the regional variation in trabecular ultrastructure across axial slices of the distal radius, which is known to be less accurate than micro CT [21]. They concluded that inner areas differ significantly from outer areas in terms of BV/TV and TbN. However, segmentation of the cortex was threshold based, which might result in higher overall values for BV/TV and TbN as remnants of the cortex adhere to the outer area.

However, the reported differences within the inner anterior and posterior area are minor. This is of major importance for our study design, as the outer area has not been subject of the presented analysis of our specimens.

On the volar side, the cortex with the adjacent outer area was opened with an awl and therefore not available for analysis. On the dorsal side, the cortex was not perforated with the trephine to preserve the extensor tendons, excluding the outer dorsal area for ultrastructural analysis.

Our results represent pooled data from all patient specimens irrespective of defect size, age or gender. Further analysis of our results did not reveal any correlations between any of these factors and the bone quality of the defect zone after implant removal, as compared to the unaffected bone. A possible explanation for this may be based on the small sample size and heterogeneity of the study group. In addition, a wide distribution between different patients was found for the ratio of architectural parameters from the dorsal to volar aspect. While complete trabecular regeneration was found in certain specimens, in other specimen, the bone ultrastructure in the dorsal aspect was still reduced by up to 50%, compared to the volar aspect.

The small sample number and heterogeneity of the specimens in our study therefore represent a weakness posing certain limitations for conclusions from the statistical analysis. In the light of this heterogeneity, further studies are warranted to determine the impact of age, gender and bone biology on trabecular bone regeneration.

However, this is, to our knowledge, the first attempt of a quantitative ultrastructural analysis of metaphyseal bone defects in humans after indirect reduction and locking plate application. Therefore, all patients who agreed in specimen harvest during implant removal were included irrespective of age and gender. Patients 75 years and older are missing in this particular study because most of these patients are treated conservatively. In case of an operative procedure, implant removal is usually not performed in this age group.

While the application of autologous bone graft was the treatment of choice for many years for the filling of the bone voids created by osteosynthesis of distal radius fractures with conventional plating techniques, it has been argued that modern fixation techniques using locking plates could be used without bone graft or substitutes, while still providing an environment for sufficient bone regeneration. Our results demonstrate that there is insufficient evidence to warrant any generalized recommendations against the filling of the defect void by autologous bone grafts or substitutes. We therefore argue that the decision to use fillers or not has to rely on the surgeon’s clinical experience, until sufficient evidence is collected to establish clear guidelines which metaphyseal defects rely on additional treatment.

## References

[1] Claes L, Veeser A, Gockelmann M, Simon U, Ignatius A (2009). A novel model to study metaphyseal bone healing under defined biomechanical conditions. Arch Orthop Trauma Surg.

[2] Walsh WR, Chapman-Sheath PJ, Cain S, Debes J, Bruce WJ, Svehla MJ (2003). A resorbable porous ceramic composite bone graft substitute in a rabbit metaphyseal defect model. J Orthop Res.

[3] McBirnie J, Court-Brown CM, McQueen MM (1995). Early open reduction and bone grafting for unstable fractures of the distal radius. J Bone Joint Surg (Br).

[4] Leung KS, Shen WY, Leung PC, Kinninmonth AW, Chang JC, Chan GP (1989). Ligamentotaxis and bone grafting for comminuted fractures of the distal radius. J Bone Joint Surg (Br).

[5] Bone LB (1987). Fractures of the tibial plafond. The pilon fracture. Orthop Clin North Am.

[6] Koval KJ, Helfet DL (1995). Tibial plateau fractures: evaluation and treatment. J Am Acad Orthop Surg.

[7] Pechlaner S (1993). Distal intra-articular radius fractures. Indications for and technique of open reduction and plate osteosynthesis. Orthopade.

[8] Ruwe PA, Randall RL, Baumgaertner MR (1993). Pilon fractures of the distal tibia. Orthop Rev.

[9] Strickler M, Nagy L, Buchler U (2001). Rigid internal fixation of basilar fractures of the proximal phalanges by cancellous bone grafting only. J Hand Surg (Br).

[10] Haidukewych GJ (2004). Innovations in locking plate technology. J Am Acad Orthop Surg.

[11] Figl M, Weninger P, Jurkowitsch J, Hofbauer M, Schauer J, Leixnering M (2010). Unstable distal radius fractures in the elderly patient–volar fixed-angle plate osteosynthesis prevents secondary loss of reduction. J Trauma.

[12] Monfoulet L, Rabier B, Chassande O, Fricain JC (2010). Drilled hole defects in mouse femur as models of intramembranous cortical and cancellous bone regeneration. Calcif Tissue Int.

[13] Uhthoff HK, Rahn BA (1981). Healing patterns of metaphyseal fractures. Clin Orthop Relat Res.

[14] Uusitalo H, Rantakokko J, Ahonen M, Jamsa T, Tuukkanen J, KaHari V (2001). A metaphyseal defect model of the femur for studies of murine bone healing. Bone.

[15] Flinkkila T, Nikkola-Sihto A, Raatikainen T, Junila J, Lahde S, Hamalainenn M (1999). Role of metaphyseal cancellous bone defect size in secondary displacement in Colles’ fracture. Arch Orthop Trauma Surg.

[16] Parfitt AM, Drezner MK, Glorieux FH, Kanis JA, Malluche H, Meunier PJ (1987). Bone histomorphometry: standardization of nomenclature, symbols, and units. Report of the ASBMR Histomorphometry Nomenclature Committee. J Bone Miner Res.

[17] Gerich T, Blauth M, Witte F, Krettek C (2001). Osteosynthesis of fractures of the head of the tibia in advanced age. A matched-pair analysis. Unfallchirurg.

[18] Schultze-Mosgau S, Keweloh M, Wiltfang J, Kessler P, Neukam FW (2001). Histomorphometric and densitometric changes in bone volume and structure after avascular bone grafting in the extremely atrophic maxilla. Br J Oral Maxillofac Surg.

[19] Mueller TL, Van Lenthe GH, Stauber M, Gratzke C, Eckstein F, Muller R (2009). Regional, age and gender differences in architectural measures of bone quality and their correlation to bone mechanical competence in the human radius of an elderly population. Bone.

[20] Braunstein V, Duda S, Sprecher CM, Brighenti V, Arora R, Tami A (2011). Comparison of regional distribution of cancellous bone in osteoporotic and non-osteoporotic distal radii. Unfallchirurg.

[21] Sode M, Burghardt AJ, Kazakia GJ, Link TM, Majumdar S (2010). Regional variations of gender-specific and age-related differences in trabecular bone structure of the distal radius and tibia. Bone.

